# Physical activity induced alterations of gut microbiota in humans: a systematic review

**DOI:** 10.1186/s13102-022-00513-2

**Published:** 2022-07-07

**Authors:** Hanna Dziewiecka, Harpal S. Buttar, Anna Kasperska, Joanna Ostapiuk–Karolczuk, Małgorzata Domagalska, Justyna Cichoń, Anna Skarpańska-Stejnborn

**Affiliations:** 1 Department of Biological Sciences, Faculty of Physical Culture in Gorzow Wielkopolski, Poznan University of Physical Education, Estkowskiego 13, 66-400 Gorzów Wielkopolski, Poland; 2 Department of Pathology & Laboratory Medicine, Faculty of Medicine, University of Ottawa, Ottawa, ON K1H 8M5 Canada

**Keywords:** Gut microbiota, Athletic performance, Physical activity, Gut permeability, Leaky gut, Microbiota composition, Microbiota diversity, Exercise

## Abstract

**Background:**

Gut microbiota is considered to have a great impact on human health and disease. While it is widely recognized that the gut microbiota of healthy individuals differs from those with obesity, inflammatory bowel disease, metabolic syndrome, and other chronic diseases, the alterations of gut microbiota with physical activity are not fully understood. Accordingly, we performed this systematic review to address the question regarding the effects of mild and intense exercise on the gut microbiota in humans.

**Methods:**

The comparative analyses of gut microbiota were conducted following the PRISMA protocol to determine the differences in the active vs. non-active individuals (phenotypes) (*n* = 11), including the influence of physical activity intervention on the human gut microbiota (*n* = 13); the differences in the gut microbiota of athletes vs. non-athletes (*n* = 8); and the microbiota status at different stages of athletic performance or intervention (*n* = 7), with various of physical activities, sport disciplines, and activity duration. Literature searches were completed using four databases: PubMed, Web of Science, Scopus, and EBSCO, and 2090 articles were retrieved by using appropriate keywords. The low heterogeneity of the studies hasn’t allowed us to prepare a meta-analysis. After excluding 2052 articles, we ultimately selected 38 articles that met the eligibility criteria for this review.

**Results:**

The data analyses revealed that in non-athletes rising physical activity markedly influenced the relative abundance of short-chain fatty acid (SCFA). Aerobic training that lasted 60 min, and physical activity that characterized 60% HRmax or more also influenced beta diversity indexes. The results showed that athletes harbor a more diverse type of intestinal microflora than non-athletes, but with a relatively reduced abundance of SCFA- and lactic acid-producing bacteria, thereby suggesting an adverse effect of intense exercise on the population of gut microbiota.

**Conclusion:**

It is concluded that the level of physical activity modulates the gastrointestinal microbiota in humans. For a long period, increasing the intensity and volume of exercise may lead to gut dysbiosis. Perhaps, proper supplementation should be considered to keep gut microbiota in large biodiversity and richness, especially under unfavorable gut conditions associated with intense exercise.

***Trial registration*:**

Prospero CRD42021264064.

## Background

Human gut bacteria consist of mainly *Firmicutes* (60–80% of all gut bacteria), and *Bacteroidetes* (20–40%), as well as a small amount of *Proteobacteria* and *Actinobacteria*, but their relative abundance varies with anatomical location among individuals. While the composition of the gut microflora can change rapidly with antibiotic use, diet, and other environmental factors, the population remains a relatively stable [1]. The physiological balance between the host and the gut microbiota has a major bearing on the host’s health [1]. In fact, the host needs the gut microbiota to support various functions of the gut: nutrient metabolism, mutagen and carcinogen neutralization, development and function of the immune system, protection from pathogens, enterocyte and intestinal epithelium development, and short-chain fatty acid (SCFA) production. SCFAs initiate enterocyte proliferation and mucin secretion, which greatly impact the tightness of the intestinal barrier. SCFAs are produced by bacteria from the genera *Clostridium*, *Eubacterium*, *Fusobacterium*, *Butyrivvibrio, Megasphera*, *Roseburia*, *Feacalibacterium*, and *Eubacterium* [2]. The composition of the microbiota, especially the presence of the above-mentioned bacteria, influences the permeability of the toxic metabolites from the gut barrier.

According to published studies, moderate exercise has a beneficial effect on intestinal permeability, absorption and assimilation of electrolytes and nutrients, and on the rate of excretion of toxic metabolic products [3]. By contrast, increasing the training load (i.e., extending the exercise time or increasing the intensity of physical exertion) may negatively affect the digestive system, and cause symptoms, such as abdominal pain, colic, flatulence, nausea, vomiting, or diarrhea. In this context, several normal physiological responses to exercise that disrupt and affect the integrity and function of the gastrointestinal (GI) tract are called “exercise-induced gastrointestinal syndrome” [4]. This syndrome is thought to affect 70% of athletes and occurs 1.5 to 3-times more often among qualified athletes than among amateurs [4]. It follows two distinct pathways: cardio–gastro–intestinal and neuroendocrine–gastro–intestinal signal pathways. The former causes redistribution of the blood flows to the working muscle and peripheral circulation, while the latter is associated with increased sympathetic activation and the resulting decrease in the functional capacity of the gastrointestinal tract [4]. Camilleri [5] suggested that physical exercise may disturb the immune system of the digestive tract (i.e., damage the lumen of the digestive tract), which may result in an increased inflammatory response and gastrointestinal symptoms [5]. Further, Camilleri proposed that changes in the composition of the intestinal microbiota, characterized by an increase in its alpha diversity and the abundance of several dominant bacteria, such as *Bacteroides*, increase intestinal permeability [5]. Published literature shows the occurrence of acute and chronic diseases, not only in the digestive system, is associated with alterations in the composition of the intestinal microflora [6, 7]. "Dysbiosis" is the loss of commensal bacteria with possible beneficial metabolic activity and the overgrowth of opportunistic pathogens, as well as reduced biodiversity [6, 7].

In this review, we will try to answer the question: how much physical effort is healthy for the human gut microbiota? We have scrutinized all published manuscripts on mild and vigorous physical activity on the population of microbiota, regardless of the size of participants. However, still, a small amount of research has been done on the changes in the microbiota in athletes. Especially, a small number of manuscripts were found about extreme physical effort and at various stages of training. There are some manuscripts about case studies because knowledge of extreme physical activity is still very low. We are cognizant of the fact that this is not the first review about the influence of physical activity on the population of microbiota [8–13], there were even systematic reviews. In our review, we decided to condense articles no matter the study design, amount of the samples, or methods used to measure microbiota. From our practice we know that it is very hard to assemble a research group of high amounts of highly trained athletes, that’s why we decided to accept all manuscript and supplements the knowledge from previous reviews on this topic, in particular general physical activity. Among published reviews Aya et al. [14] in the systematic review focused on cross-sectional studies, Dorelli et al. [15] used study designs with a control group that was measured only by the 16S rRNA method, Mitchel et al. [16] collected data from rodents, large animals and humans, Ortiz-Alvares et al. [17] concentrated on different length of exercise periods, Shahar et al. [18] paid attention to interventions that last at last five weeks, Zheng et al. [19] condensed knowledge about the influence of exercise on obesity and type 2 diabetes, Cataldi et al. [20] excluded works without control groups, Clark et al. [21] focused on the gut-brain axis, whereas Clemente et al. [22] collected data about aerobic or aerobic combined with resistant training only. Therefore, knowledge gained in this review will enrich the current knowledge about the consequences of various physical activities on gut microbiota. In analyses, we have examined the differences in the gut microbiota of active vs. non-active individuals (phenotypes); the influence of physical activity intervention on the human gut microbiota; the differences in gut microbiota among athletes vs. non-athletes; and the microbiota status at different stages of athletic performance or intervention.

## Methods

### Literature search strategy

The current study is a systematic review of literature focusing on the effect of training load on the gut microbiota. The systematic review followed the PRISMA *(*Preferred reporting items for systematic reviews and meta-analyses) protocol and was registered in PROSPERO, the International Prospective Register of Systematic Reviews, under the registration number CRD42021264064. Four databases were searched: PubMed, Web of Science, Scopus, and EBSCO (Elton Bryson Stephens Company).

The literature search included original papers written in English and published before 17 June 2021. No year restriction was applied. The following index terms were used: “gut microbiota”, “composition”, “exercise”, and “physical activity”; all words were searched in all fields. Papers were browsed using only these keywords to broaden the search.

#### Inclusion and exclusion criteria

After the database searches, the following inclusion criteria were applied: articles in the English language, studies involving males and/or females, adults, and studies evaluating physical effort on the composition of gut microbiota. The following exclusion criteria were adopted: children, subjects with disease (s), animal model studies, studies evaluating parameters other than physical effort or exercise, review papers, and meta-analysis.

#### Data extraction and study design

Data were first evaluated by three investigators (H.D., A.K., and M.D.) and then checked independently by two other investigators (A.S.-S., and J.O.-K.). First, all articles retrieved using the keyword search were downloaded. Then, all replicates were removed, and article abstracts were analyzed using the eligibility criteria. Finally, the whole text of articles that met the eligibility criteria (*n* = 38) was reviewed. Manuscripts referring to the children were not taken under review because it has been observed that the microbiota of children at age of 3 years old in 40–60% is similar to the microbiota of healthy adults. Moreover, children achieve in adolescence a microbiota composition comparable to that of adults [23]. Each publication selected for review was critically evaluated for inclusion in this review. If the full text of a publication was not publicly available, then its author was contacted for a pdf copy.

The publications were grouped in this manner to facilitate data interpretation. Only data on the influence of physical exercise on the gut microbiota of adults were extracted for review. The articles selected for this review were divided into four groups for the following analyses (one article was used twice), athletes were separated from non-athletes due to the different adaptations to the physical effort, training loads, and diet [9]:

1. Differences in the microbiota of active and nonactive individuals (phenotypes) (*n* = 11),
2. Differences in the gut microbiota of athletes and non-athletes (*n* = 8),
3. Microbiota status in athletes at different stages of preparation or intervention (*n* = 7),
4. Influence of physical activity intervention on the human gut microbiota (*n* = 13).

#### Quality assessment

Following the analysis described in subsection Methods, the evidence level was assessed by three independent reviewers (H.D., A.K., and M.D.) using the 2011 method of the Oxford Centre for Evidence-Based Medicine (OCEBM), developed by an international group of investigators considering feedback from clinicians, patients, and researchers. The OCEBM method allows rapid identification of the likely best evidence encouraging clinicians, researchers, and patients to autonomously assess evidence [24] (Table 1).

**Table 1:** The Oxford 2011 Levels of Evidence

Evidence level (treatment benefits)
Level 1: Systematic review of randomized trials or *n*-of-1 trials
Level 2: Randomized trial or observational study with dramatic effect
Level 3: Non-randomized controlled cohort/follow-up study
Level 4: Case-series, case control study, or historically controlled study
Level 5: Mechanism-based reasoning

### Statistical analyzes

**Fig. 1:**
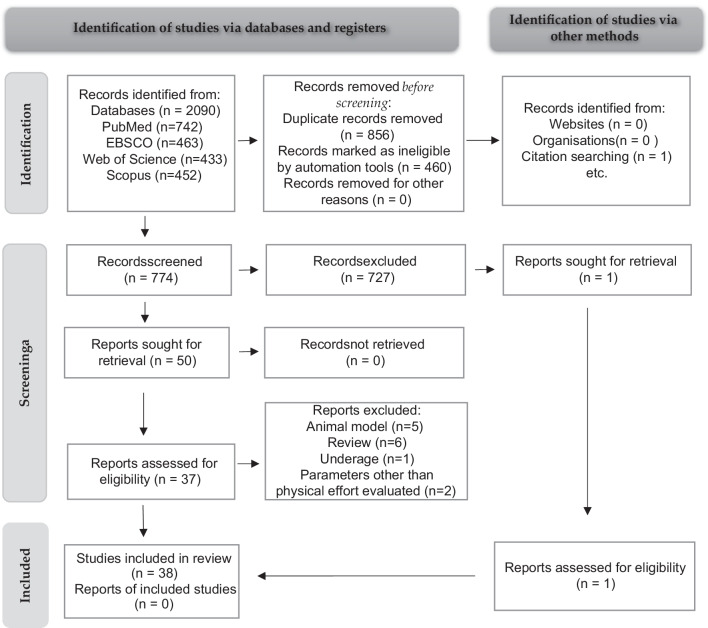
Profile of data extraction (the figure was made by the statement of PRISMA protocol [25])

A quantitative illustration using descriptive tables, without statistics, has been performed. The studies had reported data in a different format or/and study design. Summary tables were filled with information from each study, including physical activity, investigation period, characteristics of participants, and outcomes (changes in gut composition). Low heterogeneity in the studies was found and a limited number of studies investigating specific physical activity. Therefore, it was not possible to extract data for a meta-analysis for statistical comparison (Fig. 1).

## Results

The literature search identified 2090 potential articles. After the removal of 856 duplicates, and 460 records marked as ineligible by automation tools, 774 records underwent title and abstract screening. Full texts of 50 articles were evaluated, and 38 articles were included in the review (one came from citation). Results are summarized in four tables:

i. Table 2 shows differences in the microbiota of active and non-active individuals.
ii. Table 3 shows differences in the gut microbiota of athletes and nonathletes.
iii. Table 4 depicts microbiota status among athletes at different stages of preparation or interventions.
iv. Table 5 shows the influence of physical activity intervention on the human gut microbiota.

**Table 2:** Changes in the gut microbiota (phenotypes) depend on the level of physical activity

OCEBM/Study design	Age (yrs.)	Study group	Method of fecal microbiota analysis	Diversity indexes, *F/B* ratio	Fecal results of group with high level of physical activity				Reference
					Phylum/order	Family	Genus	Species	
Level 2/Cross—sectional	18–40	Premenopausal women, *N* = 40	sequencing analysis 16S rRNA (V3, V4 region)	→ Alpha diversity (Chao1, Shannon) ≠ Beta diversity (PCoA) → F/B	Phylum: no differences	*	↑*Bifidobacterium* ↑*Coprococcus* ↑*Paraprevotella* ↑ *Ruminococcacea unclassified1* ↓*Odoribacter* ↓*Turicibacter* ↓*Ruminococcacea unclassified2*	↑*Akkermansia muciniphila* ↑*Faecalibacterium prautznnii* ↑*Roseburia hominis*	[26]
Level 2/Cross—sectional	25.7 ± 2.2	Healthy adults (F, M), *N* = 37	sequencing analysis 16S rRNA	↑F/B	*	*	*	VO_2max_ explained 22% of variance of individual gut bacteria	[27]
Level 2 /Cross—sectional	> 65	Older healthy adults (F, M), *N* = 207	sequencing analysis 16S rRNA (V3 region)	↑Alpha diversity (Observed Species) ↑Beta diversity (Bray–Curtis)	Order: ↑*Bifidobacteriales* ↑*Clostridiales*	*	*	*	[28]
Level 2 /Cross—sectional	18–35	Healthy adults (F, M), *N* = 39	sequencing analysis 16S rRNA (V3, V4 region)	↑Alpha diversity (Chao, Shannon, Simpson) ≠ Beta diversity (Bray–Curtis)	*	*	↑*Adlercreutzia* ↑*Coprococus* ↑*Roseburia* ↑Unknown members of *Clostridiales*	*	[29]
Level 2/ Cross—sectional	69–76, 72 average	Seniors (M, F), senior orienteering athletes (*n* = 28), community-dwelling older adults (*n* = 70), *N* = 98	whole genome sequencing (WGS)	→ Alpha diversity (Shannon) » Beta diversity (Jaccard, Unifrac)	*	*	*	↑*Feacalibacterium prausnitzii* ↓*Bilophila* unclassified ↓*Parasaturella excrementihominis*	[30]
Level 2/ Cross—sectional	22.5 ± 2.9	Students, *N* = 140	sequencing analysis 16S rRNA	→ Alpha diversity (Shannon) → F/B	*	*	↓*Dialister* ↓*Lachnobacterium* ↓*Megasphera* ↓*Paraprevotella*	*	[31]
Level 2/ Cross—sectional	78–98, 84 average	Older healthy adults (M), *N* = 373	sequencing analysis 16S rRNA (V4 region)	→ Alpha diversity (Shannon) » Beta diversity (Unifrac)	*	*	↑*Cetobacterium* ↓*Coprobacillus* ↑*Feacalibacterium* ↑*Streptophyta* ↑*Clostriudium* ↑*Lachnospira* ↑*Prevotella* ↓*Aldercreutzia* ↓*Alistipes* ↓*Anaerotruncus* ↓*CC-1115* ↓*Clostridia SHA-98* ↓*Megasphera*	*	[32]
Level 2/ Cross—sectional	18 ± 0.6	Students (F, M), N = 373	sequencing analysis 16S rRNA (V4 region)	→ Alpha diversity (Chao1, OTU) ≠ Beta diversity (PCoA) → F/B	*	*	↑*Lachnospira* ↓*Enterobacteriales genus member*	*	[33]
Level 2/ Cross—sectional	23.1 ± 3.1	Students (F, M), *N* = 59	sequencing analysis 16S rRNA	*	Phyla: ↓*Firmicutes*	*	*	*	[34]
Level 2/ Cross—sectional	19–49	Premenopausal women, *N* = 71	sequencing analysis 16S rRNA	*	*	*	↑*Bacteroides* ↓*Enterobacteria*	↓*Eubacterium rectale* ↓*Clostridium coccoides*	[35]
Level 2/ Cross—sectional	> 61	Older healthy adults, *N* = 897	sequencing analysis 16S rRNA	→ Alpha diversity (Shannon)	*	↑*Bacteroidaceae* ↑*Campylobacteraceae* ↑*Clostridiaceae* ↑*Corynebacteriaceae* ↑*Fusobacteriaceae* ↑*Paraprevotellaceae* ↑*Peptostreptococcaceae* ↑*Turicibacteraceae* ↓Actinomycetaceae ↓Barnesiellaceae ↓Desulfovibrionaceae ↓Oxalobacteraceae ↓Pseudomonadaceae ↓S24‐7			[36]

**Table 3:** Changes in the gut microbiota depend on the type of physical activity in athletes

OCEBM/ Study design	Age (yrs.)	Study group	Method of fecal microbiota analysis	Diversity indexes, *F/B* ratio	Fecal results of group with high level of physical activity				Reference
					Phyla	Family	Genus	Species	
Level 2/Cross – sectional (only data that characterized adult elites)	19–26	Rowers (F): adult elite athletes *N* = 7	sequencing analysis 16S rRNA (V3, V4 region)	↑Alpha diversity (Shannon, Simpson) » Beta diversity (Jaccard, Unifrac), ↑F/B	↑*Firmicutes*	*	↑*Clostridiales* unclassified ↑*Feacalibacterium* ↑*Lachnospiraceae* unclassified ↑*Ruminococcaceae* unclassified ↓*Prevotellla*	*	[37]
Level 2/Cross—sectional	19–28	Bodybuilders (*n* = 15), elite runners (*n* = 15), control group (*n* = 15) (M), *N* = 45	sequencing analysis 16S rRNA (V3, V4 region)	Diversity between groups: → Alpha diversity (Chao1) ≠ Beta diversity (PCoA)	*	*	↑*Clostridium* ↑*Eisenbergiella* ↑*Faecalibacterium* ↑*Haemophilus* ↑*Sutterella* ↓*Bifidobacterium* ↓*Parasutterella*	↓*Bifidobacterium adolescentis,* ↓*Bifidobacterium longum* group*,* ↓ *Lactobacillus sake* group ↓*Blautia wexlerae,* ↓*Eubacterium hallii*	[38]
Level 2/Cross—sectional	20–24	Martial arts athletes (F, M), two competition levels [12 higher – level and 16 lower-level athletes], *N* = 28	sequencing analysis 16S rRNA (V3, V4 region)	↑Alpha diversity (Shannon, Simpson)	*	↑*Porphyromonadaceae* ↓*Veillonellaceae*	↑*Bilophila* ↑*Oscillibacter* ↑*Parabacteroides* ↑*Phascolarctobacterium* ↓*Megasphaera*	*	[39]
Level 2/Observational study	34.4 ± 3.5	Marathon runners (F, M) (*n* = 14), cross-country skiers (F, M) (*n* = 11), sedentary controls (F, M) (*n* = 46), *N* = 71	sequencing analysis 16S rRNA	↑Alpha diversity (Shannon, Simpson, Chao1), ↑F/B	*	*	↑*Prevotella* Marathon runners only: Genus: ↑*Veilonella*	*	[40]
Level 2/Randomized control intervention trial	Elite athletes: 30.0 ± 9.9 Control group: 33.4 ± 7.9	Elite athletes (mainly cyclists and triathletes) (F, M) (*n* = 13), control group (F, M) (*n* = 11), *N* = 24	sequencing analysis 16S rRNA (V1, V2 regions)	→ Alpha diversity (Inverse Shannon, Chao1) ≠ Beta diversity (Bray–Curtis)	*	*↑Ruminococcaceae*	↑*Coprococcus* ↑*Parasutterella* ↓*Dialister* ↓*Odoribacter* ↓*Phascolarctobacterium*	*	[41]
Level 2/Cross—sectional	19–49	Cyclists training (for at least 2 years), *N* = 33	metagenomic whole genome shotgun sequencing (mWGS) and RNA sequencing (RNA-Seq)	*			↑*Prevotella*	↑*Methanobrevibacter smithii*	[42]
Level 2/Cross—sectional	27 ± 5	Different sports classification groups (F, M) (*n* = 9 groups, 17 different disciplines); sports classification based on peak static and dynamic components, *N* = 37	sequencing analysis 16S rRNA	Moderate dynamic component that includes sports, such as fencing				↑*Streptococcus suis* ↑*Clostridium bolteae* ↑*Lactobacillus phage* ↑*Anaerostipes hadrus*	[43]
				High dynamic and low static components, including sports, such as field hockey				↑*Bifidobacterium animalis* ↑*Lactobacillus acidophilus* ↑*Prevotella intermedia* and ↑*Feacalibacterium prausnitzii*	
Level 2/Cross—sectional	29 ± 3	Rugby players (M) (*n* = 40), and two control groups: < 25 BMI (*n* = 23), and > 25 BMI (*n* = 23), *N* = 86	sequencing analysis 16S rRNA (V4 region)	Rugby players in comparison with both control groups: ↑Alpha diversity (Shannon) »Beta diversity (Bray–Curtis)	↓*Bacteroidetes* *↑ Firmicutes*	*↑Akkermansiaceae*, *↑Ruminococcaceae,* *↑Succinivibrionaceae,* ↑*Erysipelotrichaceae* ↑*Prevotellaceae* ↑*Succinivibrionaceae* *↓Lactobacillaceae,*	*↑Succinivibrio* *↑S24-7, RC9 gut group* ↑*Prevotella* ↑*Succinivibrio* ↑*S24-7* *↓Bacteroides* *↓Lactobacillus*	*↑Akkermansia muciniphila*	[44]

**Table 4:** Microbiota status among athletes during sports preparation or interventions

OCEBM/ Study design	Age (yrs.)	Study group	Physical characteristics	Method of fecal microbiota analysis	Diversity indexes,* F/B* ratio	Results after the intervention				Reference
						Phylum	Family	Genus	Species	
Level 2/Cross – sectional	20.7 ± 3.2	Competitive middle-distance runners F (*n* = 6) M (*n* = 8), *N* = 14	3 weeks of normal training 3 weeks of high-volume training 1-week taper	sequencing analysis 16S rRNA (V3, V4 region)	→ Alpha-diversity (Shannon Index, Chao1)		↓*Pasteurellaceae*	↓*Haemophilus*	↑*Ruminococcus callidus* ↓*Haemophilus parainfluenzae* ↓*Streptococcus parasanguinis*	[45]
Level 4/Case study	32	World-class ultramarathon runner, *N* = 1	All stages of sports preparation	sequencing analysis 16S rRNA (V4 region)	↑Alpha diversity (Shannon) ↑F/B			↑*Haemophilus* ↑*Streptococcus* ↑*Veilonella* ↓*Alloprevotella* ↓*Subdolingranulum*		[46]
Level 2/Observational study	18—24	Swimmers (F, M), *N* = 13	Subjects recorded their total daily swimming yardage and the duration of daily practice	16S rRNA (V4 region)	Increase of training volume: ↑Alpha diversity (Shannon, Simpson) » Beta diversity (Bray–Curtis) ↑F/B		Increase of training volume: ↑*Bacteroidaceae* ↑*Lachnospiraceae* ↑*Ruminococcaceae*	Decrease of training volume: ↓*Coprococcus* ↓*Faecalibacterium*		[47]
Level 4/Case study	26 ± 3	Rowers (M), *N* = 3	33 days, distance 5000 km	Shotgun sequencing	↑Alpha diversity (Shannon)				↑*Dorea longicatena* ↑*Prevotella copri* ↑*Roseburia hominis* ↑*Subdoligranulum* unclassified ↓*Bacteroides finegoldii*	[48]
Level 1, Randomized control trial	> 18	Soldiers (F, M), *N* = 73	4 days country-ski march, military training	sequencing analysis 16S rRNA (V3, V4 region)	↑Alpha diversity (Shannon) → Alpha-diversity (Chao1, OTU) ↑F/B			↑*Acidaminococcus* ↑*Fusobacterium* ↑*Peptoniphilus* ↑*Peptostreptococcus* ↑*Staphylococcus* ↓*Bacteroides* ↓*Collinsella* ↓*Faecalibacterium* ↓*Roseburia*		[49]
Level 2/Single arm trial	18–50	Cross-country athletes (M), *N* = 40	Subjected to physical exertion until refusal, analysis before and after exertion	sequencing analysis 16S rRNA (V3, V4 region)	→ Alpha diversity (Shannon, OTU) ≠ Beta diversity (Bray–Curtis, Jaccard, Unifrac)			↑*Blautia* ↑*Rombutsia* ↑*Ruminococcocaceae* USG-005	↑*Escherichia coli TOP48* *↓Clostridium phoceensis* *↓Ruminiclostridium 9*	[50]
Level 2/Observational study	23–54	Half-marathon runners (F, M), *N* = 20	Average period of training before the start time: 18 months; average time to finish: 115 min	sequencing analysis 16S rRNA (V3, V4 region)	After the run: → Alpha diversity ↑ OTUs	↑*Lentisphaerae* ↑*Acidobacteria*	↑*Coprococcus*_2 ↑*Collinsella* ↑*Mitsuokella* ↑*Pseudobutyrivibrio* ↑*Romboutsia*			[51]

**Table 5:** The influence of physical activity during training intervention on the human gut microbiotaTable

OCEBM/ Study design	Age (yrs.)	Study group	Time	Physical activity	Method of fecal microbiota analysis	Diversity indexes, *F/B ratio*	Fecal results after intervention				Reference
							Phylum/class/order	Family	Genera/Genus	Species	
Level 2/ Cross—sectional	20–45	Lean (BMI 22.2 ± 2.8) and obese individuals (BMI 35.71 ± 5.11) (M, F), *N* = 33	6 weeks	Progressive training 3 times a week from 30 to 60 min, 60–75% HR	sequencing analysis 1a6S rRNA (V3 region)	Obese individuals: »Beta diversity (PCoA)	*	*	↑*Feacalibacterium spp* ↑*Lachnospira spp* ↑*Lachnospiraceae* unlassified ↑*Roseburia spp*	*	[52]
Level 4/ Case study	30 or 33	Ultramarathon or triathlon (M), *N* = 2	6 months	Each individual was trained to the level necessary for participation in an endurance-based sport competition	sequencing analysis 16S rRNA	↑Alpha diversity (Shannon) »Beta diversity (PCoA)	*	*	*	Marathoner: *↑Vaillonella pervula* ↓*Agathobacter rectalis* Triathlete: *↑Akkermansia munciphila* ↑*Bifidobacterium longum* ↑*Methanobravibacter smithii* ↓*Bifidobacterium animalis*	[53]
Level 2/ Cross—sectional	18–31	Students (F, M) Cardiorespiratory exercise (*n* = 26) Resistance training (*n* = 26) *N* = 52	8 weeks	Cardiorespiratory: 3 times a week, 60 min, 60–90% HR max Resistance training: 70–85% 1RM, 3–6 sets, 6–12 repetitions	sequencing analysis 16S rRNA (V4 region)	Cardiorespiratory fitness: »Beta diversity (Bray–Curtis, Jaccard, Unifrac) Resistance training → diversity (Bray–Curtis, Jaccard, Unifrac)	*	*	*↑Prevotella* *↑Romboutsia* *↑Dialister*	*	[54]
Level 1/ Cross—sectional	18–40	Healthy adults, BMI 22–35 kg/m2 (F, M), *N* = 25	8 weeks	Exercise 3 times a week Cardiorespiratory fitness: prospective 5, 7, 10 RTE Resistance training: 70% RM, 3 sets, 8 repetitions, progressive load	metagenomic DNA sequencing	→ Alpha diversity (Shannon) ≠ Beta diversity (Bray–Curtis)	*	*	*	*	[55]
Level 1/ Cross—sectional	20–45	Overweight and obese individuals (F, M), Control *n* = 14, Cycling to work *n* = 19, Exercise I *n* = 31, Exercise II *n* = 24) BMI 25–35 kg/m^2^, *N*—88	6 months	Cycling to work: Exercise I: 50% VO_2peak-reserve_ Exercise II: 70% VO_2peak-reserve_ 5 times a week	sequencing analysis 16S rRNA	Exercise I: → Alpha diversity (Shannon) »Beta diversity (Bray–Curtis) Exercise II: ↑Alpha diversity (Shannon) 5% »Beta diversity (Bray–Curtis)	*	*	*	*	[56]
Level 2/ Cross—sectional	Soldiers23.9 ± 1.9 Control: 30.0 ± 8.8	Soldiers (*n* = 66) or healthy adults (control *n* = 38) (M), *N* = 104	8 weeks	Participants lived in the same environment for 8 weeks, ate the same food at regular intervals, and participated in similar training and sleep regimens	sequencing analysis 16S rRNA (V4, V5 regions)	→ Alpha diversity (Shannon, OTU) ≠ Beta diversity (PCoA)	*	*	*	↑*Bifidobacterium* ↑*Murdochiella* ↓*Holdemanella* ↓*Ruminococcus_2* ↓*Streptococcus* ↓*Turicibacter*	[57]
Level 2/ Cross—sectional	Endurance group 31.4 ± 8.3 strength group 29.9 ± 7.9 control group 33.4 ± 7.9	Endurance group (*n* = 13), strength group (*n* = 12), control group (*n* = 11), *N* = 36, (F, M)	6 weeks	Two groups: endurance or strength exercise, 3 times a week	sequencing analysis 16S rRNA (V1, V2 regions)	→ Alpha diversity (Chao1) ≠ Beta diversity (Bray–Curtis)	*	↑*Ruminococcaceae*	↑*Coprococcus* ↑*Parasaturella*	*	[41]
Level 1/Randomized trial	49 ± 4	Insulin resistance (F, M), (*n* = 26), *N* = 54	2 weeks	Two groups: First: 30-s exercise bouts of all cycling efforts, Second: 40–60 min of moderate intensity (60% of VO_2 peak_) All sessions were performed under supervision, 3 times a week	sequencing analysis 16S rRNA (V3-V4 region)	Both groups: → Alpha diversity (Shannon, Chao1, OTU), ↓ F/B	↑*Bacteroidetes*	*	↓*Blautia spp.* ↓*Clostridium spp* First group: ↑*Lachnospira* Second group: Genus: ↑*Faecalibacterium* ↑*Veillonella* Species: ↑*Veillonella dispar*	*	[58]
Level 2/ Nonrandomized clinical trial	> 65	Sedentary healthy older adults (F, M), Aerobic exercise *n* = 18, trunk muscle training *n* = 14, *N* = 32	12 weeks	Aerobic exercise training, brisk walking, at an intensity ≥ three metabolic equivalents Trunk muscle training 1 h weekly	sequencing analysis 16 s rRNA	*	*	*	Aerobic exercise: Genus: ↑*Bacteroides* ↓*Clostridium XIVa*	*	[59]
Level 2/ Cross—sectional	36.8 ± 3.9	Women with sedentary lifestyle, BMI > 27.5 kg/m^2^, *N* = 19	6 weeks	Training 3 times a week with supervision Endurance exercise: weeks 1 and 2: 40 min, weeks 3 and 4:50 min, weeks 5 and 6:60 min	sequencing analysis 16S rRNA (V4 region)	→ Alpha diversity »Beta diversity (Jaccard) → F/B	*↑Verrucomibrobia*	*↑Verrucomicrobiaceae,*	*↑Akkermansia* ↓*Proteobacteria*	*,* *	[60]
Level 2/ Cross—sectional	Lean 29 ± 2 Overweight 31 ± 2	Lean: fat mass 21 ± 2% (*n* = 14), Overweight: fat mass 33 ± 2% (*n* = 15), *N* = 29 (M)	3 weeks	3 weeks of h*igh intensity interval training*	sequencing analysis 16S rRNA (V3, V4 region)	→ Alpha diversity (Shannon) ≠ Beta diversity (Bray–Curtis)	*	*	*	*	[61]
Level 1/ Randomized crossover trial	62–76	Older adults (M), *n* = 16, *n* = 17, *N* = 33	5 weeks	Endurance exercise: week 1: 60% VO_2peak_, weeks 2 and 3: 70% VO_2peak_, weeks 4 and 5: 75% VO_2peak_	sequencing analysis 16S rRNA (V3, V4 region)	→ Alpha diversity (Shannon, OTU) ≠ Beta diversity (Bray–Curtis, PCoA)	*	*	↑*Oscillospira*	↓*Clostridium difficile*	[62]
Level 1/ Randomized crossover trial	60–75	Inactive older adults (F) *n* = 7, *N* = 14	8 weeks	Aerobic and resistance exercise, sessions of approximately 60 min each	sequencing analysis 16S rRNA (V4 region)	→ Alpha diversity (Shannon, Simpson, Chao1) »Beta diversity (PCoA)	↓*Firmicutes* ↑*Bacteroidetes* ↓*Clostridia* ↑*Betaproteobacteria* ↑*Burkholderiales*	↑*Acidaminococcaceae*	↑*Mitsuokella* ↑*Phascolarctobacterium*	*	[63]

We summarize articles this way to better understand the results of our review.

### Characteristics of included studies:

Table 2.

Table 3.

Table 4.

Table 5.

### Symbols/Abbreviations used:

 → , ≠ -unchanged.

↑,»-increased.

↓-decreased.

*-no data.

F/B-Firmicutes/Bacteroidetes ratio.

Alpha-diversity indexes: Chao1, Shannon, Simpson.

Beta-diversity indexes: PCoA (Principal Coordinates Analysis), Bray–Curtis, Jaccard, Unifrac.

Observed OTUs-Observed operational taxonomic unit.

VO_2_peak is the highest/maximum oxygen consumption achieved during a clinical/research graded exercise test.

VO_2_max is the maximal aerobic power defined as the maximum amount of oxygen that an individual can utilize during intense or maximal exercise.

F-female; M-male.

HR-heart rate; RM-repetition maximum; RTE-repetition time exercise.

BMI-body mass index, RM-repetition maximum, RTE–resistant.

### Differences in the microbiota of active and non-active individuals

In Table 2 total amount of manuscripts is 11, all of them are cross-sectional [26–36]. The size of the group in these manuscripts is less than 50 in 3 articles, < 50 < 150 in 4 articles, and larger than 150 in 4 articles. Moreover, besides two articles [26, 35] all articles were about both men and women. We observed changes in the gut microbiota (phenotypes) when physical activity was rising in non-athletes, mainly increases in a genus of SCFA-producers [26, 29, 30, 32, 33, 36]. Furthermore, different indexes of alfa-diversity weren’t changed [26, 30, 31, 33, 36], but in one study we can see an increase in *Akkermansia muciniphila* [26].

### Differences in the gut microbiota of athletes and non-athletes

In Table 3 we have 8 articles [37–44], 6 of them were cross-sectional, 1 observational, and 1 randomized control interventional trial. The sample size oscillated from 7 to 73 subjects. Characteristics of the group were very various: martial arts athletes, rugby players, triathletes, runners, and bodybuilders, in the aged 19 to 49. A highly trained athlete’s microbiome can be described as a microbiome that has a high alpha-diversity [37, 39, 40, 44]. Changes in bacteria family, genus, and species differ a lot among the groups.

### Microbiota status in athletes at different stages of preparation or intervention

In Table 4 there are 7 articles [45–51] with different study designs: 1 cross-sectional, 2 case studies, 2 observational, 1 randomized control trial, and 1 single-arm trial, amount of the group oscillates from 1 to 73. Participants' age was between 18 and 54, only 3 studies focused on men, and others considered both sexes. The sports preparation or intervention varies a lot among the studies, from 4-day country ski military training, which was the largest sample (*N* = 73), to the highest intensity and volume of a word-class marathon runner (*N* = 1). The diversity indexes outcomes and changes in family, genus, and species of bacteria are not easy to compare, because of the various groups included in this table.

### Influence of physical activity intervention on the human gut microbiota

Table 5 contains the highest amount of research articles [41, 41, 52–63], 8 are cross-sectional, 1 is a case study, 3 are randomized control trials, and 1 is a non-randomized clinical trial. The sample size oscillates from 2 to 104, 4 studies are about men only, 2 are about women only, rest are about both sexes. The duration of exercise intervention varies from 2 weeks to 6 months. Exercise intervention hasn’t influenced alfa and beta diversity but it had an impact on SCFA producers [41, 52, 54, 58], in two studies genus *Akkermansia* and species *Akkermansia muciniphila* occurred [53, 60]. Aerobic and resistant training together [63] or only endurance exercises [60] that lasted 60 min had an impact on beta diversity indexes. Moreover, physical activity that characterized 60% HRmax [52, 54] or more also influenced beta diversity indexes. What is interesting, exercises with 70%VO_2peak_ influenced the alfa diversity [56] or decreased *Clostridium Difficile* [62].

## Discussion

### Diversity of the human gut microbiota

Diversity and richness are among the major parameters describing the human gut microbiota. Identification of dissimilarities in microbial diversity in different populations, for example, smokers vs. nonsmokers and ill vs. healthy, is a fundamental step of microbiome studies. For instance, reduced microbial diversity is associated with various host phenotypes, such as obesity, fatty liver disease, type II diabetes, and inflammatory bowel disease, to name a few. Clinical interventions (e.g., antibiotic use) and environmental factors (e.g., diet, smoking, and physical activity) also affect the microbial diversity [64]. Accordingly, biodiversity (alpha diversity Shannon Index) parameters have been compared in athletic activity, and exercise studies. The microbial diversity was reported as unchanged regardless of the level of physical activity in five studies [26, 30–32, 36], while it was reportedly increased with increased physical activity in two studies [28, 29]. Although the diversity of gut microbiota of athletes was reported to be higher than that of nonathletes in four studies [37, 39, 40, 44]. In the current review, the diversity parameters did not respond to the stimulus of exercise in non-training individuals [55, 57, 58, 60, 61] but were affected by the training load in highly trained athletes [46–48]. Therefore, the microbial diversity does not appear to be related to the physical exercise as per se, but to the appropriate “intervention”, i.e., the time or intensity of the physical effort. These conclusions are supported by studies in the rat model conducted by Allen et al. [65], who showed that forced vs. voluntary training differently impacts the gut microbiota composition. In addition, Grosicki et al. [46] analyzed changes in the intestinal microbiota at all stages of an athlete's preparation for an ultramarathon. They observed the highest alpha-diversity values during the training periods of the lowest intensity (the preparation period and post-start period), with the lowest values reported upon an increase of the physical effort load (the pre-start period) and immediately after the physical performance, i.e., the recovery period. Furthermore, Karl et al. [49] showed that greater microbiota alpha diversity is not always related to gut health but may be associated with the growth of potentially harmful bacteria. This is supported by an increased abundance of the potentially pathogenic genus *Veillonella* [64, 66–68] in the gut of marathon runners [40, 46, 53]. Although Sheiman et al. observed an increase in *Veillonella* relative abundance in marathon runners post-marathon and isolated a strain of *Veillonella atypica* from stool samples. Inoculation of this strain into mice significantly increased exhaustive treadmill run time probably because *Veillonella* utilizes lactate as their sole carbon source [69].

### Changes in Firmicutes and Bacteroidetes abundance in the gut

*Firmicutes* and *Bacteroidetes* are the two most abundant phyla that inhabit the human gut. According to some reports, these bacteria account for up to 90% of the gut microbiota [2, 70]. The *Firmicutes* family contains several thousand species of highly diverse bacteria*. Bacteroidetes* are involved in food digestion, signal transmission, gut environment control, and inhibiting the growth of undesirable microorganisms in the gut [2]; however, their high abundance is associated with poor microbiota with low diversity [2]. Although, as mentioned earlier in Sect. 4.1, high alpha diversity is not always associated with a healthy gut [49]. Only three studies reported increased *Bacteroidetes* abundance after exercise [58, 60, 63]. Further, an increase in the *Firmicutes/Bacteroidetes* ratio is reported in six studies [27, 37, 40, 46, 47, 49], mainly among athletes. In recently reported studies, the increased ratio is associated with the obesity [71, 72]. However, the increased ratio in this particular group of microbiota can be explained by efficient energy extraction from food [73, 74], which is necessary for heavy physical exertion.

### Changes in SCFA producer abundance in the gut

Bacteria from the *Clostridium* genus are major SCFA producers. They are also involved in the pro-inflammatory immune response [75]. An increase in the relative abundance of *Clostridium* genus upon physical activity was reported in two studies: one by Jang et al. [38], who compared the gut microbiota of bodybuilders with that in a control group; and the other by Langsetmo et al. [32] in a large sample of elderly individuals. Two other studies reported a reduction in the relative abundance of the *Clostridium genus* upon exercise intervention [58, 59]. Further, one study reported a decrease in *Clostridium difficile* abundance upon exercise [62]. This bacterium is a major source of infectious diarrhea associated with toxin production in the host’s gastrointestinal tract [76, 77], especially in the elderly [78–80] and obese individuals [81, 82]. These observations suggest that moderate exercise has a positive effect on the abundance of *Clostridium* bacteria.

Another SCFA-producing bacterium whose relative abundance is affected by exercise is the genus *Feacalibacterium* and its representative *Feacalibacterium prausnitzii*. An increase in the population of *Feacalibacterium prausnitzii* was noted in relatively active individuals [26]. Also, the population of genus *Feacalibacterium* was compared after a moderate exercise intervention [58], and in athletes versus non-training subjects [37, 38]. A decrease in its abundance was observed in professional athletes upon extreme physical exertion [47, 49]. Numerous authors have pointed out the anti-inflammatory effect of *Feacalibacterium prausnitzii* [83, 84], as well as of the entire *Feacalibacterium* genus [85], by associating the abundance of these bacteria with the alpha diversity of microbiota [86]. Overall, the appraisal of available data suggests a positive effect of moderate exercise compared with that extreme exercise.

Another SFCA producer is the genus *Roseburia* [87, 88]. An increase in the *Rosuburia* genus and its representative *Roseburia hominis* abundance was noted in various studies when comparing an individual’s normal physical activity and upon physical exercise [26, 29, 48, 65]. A decrease in its abundance was only observed upon extreme physical exertion [49], confirming the previous observations of a negative effect of extreme exertion on the gut microbiota. Further, the enhanced population in the family of *Lachnospiraceae* or genus *Lachnospira* confirms the positive impact of moderate-intensity exercise on the gut microbiota [32, 33, 37, 47, 52, 58].

Another important SCFA producer is the genus *Coprococcus* [89, 90]. It is associated with positive outcomes in the treatment of inflammatory bowel disease [91] and a reduced risk of *Campylobacter* infection [92]. A marked increase or abundance of the *Coprococcus* genus was observed in comparative studies of active vs. inactive individuals [26, 41]. Furthermore, Hampton–Marcell et al. [47] reported a decrease in the relative *Coprococcus* abundance with a decreased physical exercise in swimmers during the starting season. Interestingly enough, the *Coprococcus_2* abundance tripled in runners after running a half-marathon [51], indicating that even extreme physical exertion can have a positive influence on the gut microbiota.

### Changes in the lactic acid producer abundance in the gut

When discussing the role of gut microbiota, the lactic acid-producing bacteria from the genus *Bifidobacterium* and *Lactobacillus*, which are widely used in probiotics [2], must be mentioned. Their positive impact on human health is well documented by [93]. When administered as probiotics, they reduce hypercholesterolemia [94], improve the parameters of diabetes mellitus [95], and regulate local and systemic immune responses [96, 97]. Further, their decreased population has been reported in individuals with severe depression [98]. In the context of the effects of exercise, an increase in their abundance was observed in one exercise intervention study [57], and in comparative studies done on athletes and non-athletes [43, 44]. By contrast, a reduction in their abundance is reported in highly trained athletes [53].

### Other types of bacteria

Exercise affects the abundance of species from the gram-negative *Prevotella* genus. An increased abundance of *Prevotella* was noticed when comparing athletes to non-athletes [40, 44]. Moreover, a higher abundance of *Prevotella* was seen during a 3300-km row in rowers [48]. When accompanied by a high abundance of *Bacteroides* and *Akkermansia muciniphila*, this bacterium is a marker of good intestinal health [70]. However, that was not the case in the above studies. When not accompanied by a higher abundance of *Bacteroides* and *Akkermansia muciniphila*, *Prevotella* is thought to support pro-inflammatory processes [99], opportunistic infections, and diseases related to intestinal dysbiosis, and are proposed to be a marker of intestinal dysbiosis [100]. These reported observations appear to confirm the negative impact of physical activity on the gut microbiota of qualified athletes.

The *Ruminaceae* family has been linked to a reduced intestinal permeability in 102 women's [101]. An increase in its abundance upon physical exercise has been noted in numerous studies [26, 37, 41, 44, 47, 50], both when considering different phenotypes and athletes, which indicates the positive effect of physical activity on these bacteria. Two important geniuses belong to this family: *Ruminococcus*, proposed by Hills et al. as a marker of intestinal dysbiosis [70]. *Ruminoccocus* genus and its representative were decreased in intervention studies [57] and during sports preparation [45]*.* The second genus that belongs *to the Ruminaceae* family is *Oscillospira*, which is closely related to human health [100] and lean individuals [102, 103]. The abundance of *Oscillospira* positively correlates with microbial diversity, high-density lipoproteins, and sleep-time duration, and is inversely correlated with blood pressure, fasting glucose levels, triglycerides, and uric acid [101]. In addition, *Oscillospira* abundance is reduced in Crohn’s disease and fatty liver disease. From the literature reviewed for the current systematic review, an increase in *Oscillospira* abundance in intervention studies was only reported by Taniguchi et al. [62].

Another bacterium, proposed as a new probiotic [104], is *Akkermansia muciniphila*, the main representative of the *Verrucomicrobia* phylum. Zhai et al.[105] consider it as a marker of a healthy gut, which is associated with lean people [86]. Although, its low abundance is observed in obese individuals and diabetics [106, 107]. That may be because the presence of *A. muciniphila* is associated with improved fat oxidation [108–110]. An increased relative *A. muciniphila* abundance was reported in relatively active people [26, 44] and after exercise intervention *Verrucomibrobia, Verrucomicrobiaceae, and Akkermansia* respond [60], confirming the notion that moderate-intensity exercise positively affects gut health.

Exercise or physical activity may represent a strong modulator of gut microbiota composition. Moreover, the gut-muscle communication in human pathophysiology may be bidirectional, with gut microbiota representing a “cross-road” among environment, and skeletal muscle [111]. The well-known positive health effects of exercise may be mediated by its beneficial modifications to the gut microbiota. However, when there is an exercise overload, these possible beneficial effects are overweighed by increased intestinal permeability and oxidative stress, promoting inflammation and a catabolic state that negatively impacts the functionality of skeletal muscle [112].

The first limitation of this review comes from the searching process even dough we proceed in the process through Prisma protocol, there is one article that comes out from the citation. That’s why there is a small possibility that we missed more than one manuscript.

The second limitation is the sample size of the groups involved in this review from 1 to 373 participants, which may influence the outcomes.

The third limitation is different methods of analyzing microbiota shot gut sequencing, whole-genome sequencing, and sequencing analysis 16S rRNA (on different regions V1, V2, V3, V4), which could also influence the data in our systematic review. Moreover, another limitation is the high diversity of the participants in the athlete’s group that were difficult to compare.

The last limitation is the limited number of studies reported on this research topic so far, and the small number of participants in the studies. This aspect is especially evident for data on high-performance athletes.

`Future direction:

- Well design, randomized exercise intervention studies are needed to access the therapeutic potential of exercise in the context of gut microbiota. The model of exercise that will be used in that studies should focus on proper intensity and duration, the universal scales should be used: VO2max, HRmax. In future research, outcomes will be easier to compare.
- Observational studies in larger samples of participants (not case studies) in highly trained athletes through every stage of athlete’s preparation are highly needed. Gained outcomes will increase the current knowledge on that theme and may be useful for practitioners: coaches, sports dietitians, and sports medicine specialists in the aspect when the gut microbiota needs special attention.
- Studies that will enable a finding of a bacterial marker in gut dysbiosis. The examination of gut microbiota is still very expensive and requires special equipment. Research on a sensitive and cheap bacterial marker of the human gut microbiota in athletes is needful.
- There is still a lack of knowledge about resistant training and its influence on gut microbiota, more studies on this subject are needed.

## Conclusion

Considering the presented evidence, we conclude that the level of physical activity modulates the population of intestinal microbiota. That was apparent in athletes compared to untrained individuals. Athletes harbor a more diverse intestinal microflora than nonathletes, but with a relatively reduced abundance of SCFA- and lactic acid-producing bacteria, which may indicate an adverse effect of intense exercise on the gut microbiota.

Based on the reviewed studies, moderate-intensity exercise does not affect the diversity of the gut microbiota but impacts its composition, with an increased abundance of SCFA and lactic acid producers, also increasing the relative abundance of *Akkermansia muciniphila* and *Oscillospira*. These observations confirm the positive impact of moderate exercise on the diversity and function of the intestinal microbiota.

Furthermore, the reviewed studies confirm the notion that intense physical activity may be detrimental to the intestinal microbiota. The exercise-induced gastrointestinal syndrome may be responsible for changes observed in the gut microbiota of athletes, and the effect of exercise on the gut microbiota appears to be much stronger than anticipated. On the other hand, moderate physical activity enhances the biodiversity and function of the microbiota. Nonetheless, this issue requires further research.

In the case of physical activity understood as an environmental issue affecting the intestinal microbiota, future research should focus on the impact of various types of activities, especially in the context of training load, intensity, or frequency of exercise. In highly-trained athletes, SCFa producers decreased and potentially pathogenic bacteria increased, allowing us to design an effective intervention (diet supplementation [70] or diet strategy [9]) to keep the gut microbiota in large biodiversity and richness, especially under unfavorable gut conditions associated with intense or vigorous exercise.

## Data Availability

The datasets used and/or analysed during the current study are available from corresponding author on reasonable request.

## Abbreviations

BMI: Body mass index
EBSCO: Elton Bryson Stephens company
F/B: Firmicutes/Bacteroidetes ratio
F: Female
GI: Gastrointestinal
HR: Heart rate
M: Male
Observed OTUs: Observed operational taxonomic unit
OCEBM: Oxford centre for evidence-based medicine
PCoA: Principal coordinates analysis.
PRISMA: Preferred reporting items for systematic reviews and meta-analyses
RM: Repetition maximum
RM: Repetition maximum
RTE: Repetition time exercise
RTE: Resistant training
SCFA: Short-chain fatty acid
VO_2_max: Maximal oxygen consumption

## References

[1] Allam-Ndoul B, Castonguay-Paradis S, Veilleux A (2020). Gut microbiota and intestinal trans-epithelial permeability. Int J Mol Sci.

[2] Panasiuk A, Kowalińska J. Mikrobiota przewodu pokarmowego. 1st ed. Warszawa: PZWL Wydawnictwo Lekarskie; 2020.

[3] Peters HP, Bos M, Seebregts L, Akkermans LM, van Berge Henegouwen GP, Bol E (1999). Gastrointestinal symptoms in long-distance runners, cyclists, and triathletes: prevalence, medication, and etiology. Am J Gastroenterol.

[4] Ribeiro FM, Petriz B, Marques G, Kamilla LH, Franco OL (2021). Is there an exercise-intensity threshold capable of avoiding the leaky gut?. Front Nutr.

[5] Camilleri M (2019). Leaky gut: mechanisms, measurement and clinical implications in humans. Gut.

[6] Marchesi JR, Adams DH, Fava F, Hermes GDA, Hirschfield GM, Hold G (2016). The gut microbiota and host health: a new clinical frontier. Gut.

[7] Picca A, Fanelli F, Calvani R, Mulè G, Pesce V, Sisto A (2018). Gut dysbiosis and muscle aging: searching for novel targets against sarcopenia. Mediat Inflamm.

[8] Marttinen M, Ala-Jaakkola R, Laitila A, Lehtinen MJ (2020). Gut microbiota, probiotics and physical performance in athletes and physically active individuals. Nutrients.

[9] Jäger R, Kerksick CM, Campbell BI, Cribb PJ, Wells SD, Skwiat TM (2017). International society of sports nutrition position stand: protein and exercise. J Int Soc Sports Nutr.

[10] Clark A, Mach N (2017). The crosstalk between the gut microbiota and mitochondria during exercise. Front Physiol.

[11] Mach N, Fuster-Botella D (2017). Endurance exercise and gut microbiota: a review. J Sport Health Sci.

[12] Hughes RL, Holscher HD (2021). Fueling gut microbes: a review of the interaction between diet, exercise, and the gut microbiota in athletes. Adv Nutr Bethesda Md.

[13] Mohr AE, Jäger R, Carpenter KC, Kerksick CM, Purpura M, Townsend JR (2020). The athletic gut microbiota. J Int Soc Sports Nutr.

[14] Aya V, Flórez A, Perez L, Ramírez JD (2021). Association between physical activity and changes in intestinal microbiota composition: a systematic review. PLoS ONE.

[15] Dorelli B, Gallè F, De Vito C, Duranti G, Iachini M, Zaccarin M (2021). Can physical activity influence human gut microbiota composition independently of diet? A systematic review. Nutrients.

[16] Mitchell CM, Davy BM, Hulver MW, Neilson AP, Bennett BJ, Davy KP (2019). Does exercise alter gut microbial composition? A systematic review. Med Sci Sports Exerc.

[17] Ortiz-Alvarez L, Xu H, Martinez-Tellez B (2020). Influence of exercise on the human gut microbiota of healthy adults: a systematic review. Clin Transl Gastroenterol.

[18] Tzemah Shahar R, Koren O, Matarasso S, Shochat T, Magzal F, Agmon M (2020). Attributes of physical activity and gut microbiome in adults: a systematic review. Int J Sports Med.

[19] Zheng C, Chen XK, Tian XY, Ma ACH, Wong SHS (2022). Does the gut microbiota contribute to the antiobesity effect of exercise? A systematic review and meta-analysis. Obes.

[20] Cataldi S, Bonavolontà V, Poli L, Clemente FM, De Candia M, Carvutto R (2022). The relationship between physical activity, physical exercise, and human gut microbiota in healthy and unhealthy subjects: a systematic review. Biology.

[21] Clark A, Mach N (2016). Exercise-induced stress behavior, gut-microbiota-brain axis and diet: a systematic review for athletes. J Int Soc Sports Nutr.

[22] Clemente F, Bravini E, Corna S, Colombo E, Sartorio F, Rinaldi C (2021). The relationship between physical exercise and gut microbiota in the human being: a systematic review. Epidemiol Prev.

[23] De Filippo C, Di Paola M, Giani T, Tirelli F, Cimaz R (2019). Gut microbiota in children and altered profiles in juvenile idiopathic arthritis. J Autoimmun.

[24] Group OLoEW. The Oxford 2011 levels of evidence. UK: Oxford Centre for Evidence-Based Medicine Oxford; 2011.

[25] Page MJ, McKenzie JE, Bossuyt PM, Boutron I, Hoffmann TC, Mulrow CD (2021). The PRISMA 2020 statement: an updated guideline for reporting systematic reviews. BMJ.

[26] Bressa C, Bailén-Andrino M, Pérez-Santiago J, González-Soltero R, Pérez M, Montalvo-Lominchar MG (2017). Differences in gut microbiota profile between women with active lifestyle and sedentary women. PLoS ONE.

[27] Durk RP, Castillo E, Márquez-Magaña L, Grosicki GJ, Bolter ND, Lee CM (2019). Gut microbiota composition is related to cardiorespiratory fitness in healthy young adults. Int J Sport Nutr Exerc Metab.

[28] Castro-Mejía JL, Khakimov B, Krych Ł, Bülow J, Bechshøft RL, Højfeldt G (2020). Physical fitness in community-dwelling older adults is linked to dietary intake, gut microbiota, and metabolomic signatures. Aging Cell.

[29] Estaki M, Pither J, Baumeister P, Little JP, Gill SK, Ghosh S (2016). Cardiorespiratory fitness as a predictor of intestinal microbial diversity and distinct metagenomic functions. Microbiome.

[30] Fart F, Rajan SK, Wall R, Rangel I, Ganda-Mall JP, Tingö L (2020). Differences in gut microbiome composition between senior orienteering athletes and community-dwelling older adults. Nutrients.

[31] Gallè F, Valeriani F, Cattaruzza MS, Gianfranceschi G, Liguori R, Antinozzi M (2020). Mediterranean diet, physical activity and gut microbiome composition: a cross-sectional study among healthy young italian adults. Nutrients.

[32] Langsetmo L, Johnson A, Demmer RT, Fino N, Orwoll ES, Ensrud KE (2019). The association between objectively measured physical activity and the gut microbiome among older community dwelling men. J Nutr Health Aging.

[33] Whisner CM, Maldonado J, Dente B, Krajmalnik-Brown R, Bruening M (2018). Diet, physical activity and screen time but not body mass index are associated with the gut microbiome of a diverse cohort of college students living in university housing: a cross-sectional study. BMC Microbiol.

[34] Valeriani F, Gallè F, Cattaruzza MS, Antinozzi M, Gianfranceschi G, Postiglione N (2020). Are nutrition and physical activity associated with gut microbiota? A pilot study on a sample of healthy young adults. Ann Ig Med Prev E Comunita.

[35] Yang Y, Shi Y, Wiklund P, Tan X, Wu N, Zhang X (2017). The association between cardiorespiratory fitness and gut microbiota composition in premenopausal women. Nutrients.

[36] Zhu Q, Jiang S, Du G (2020). Effects of exercise frequency on the gut microbiota in elderly individuals. MicrobiologyOpen.

[37] Han M, Yang K, Yang P, Zhong C, Chen C, Wang S (2020). Stratification of athletes’ gut microbiota: the multifaceted hubs associated with dietary factors, physical characteristics and performance. Gut Microbes.

[38] Jang LG, Choi G, Kim SW, Kim BY, Lee S, Park H (2019). The combination of sport and sport-specific diet is associated with characteristics of gut microbiota: an observational study. J Int Soc Sports Nutr.

[39] Liang R, Zhang S, Peng X, Yang W, Xu Y, Wu P (2019). Characteristics of the gut microbiota in professional martial arts athletes: A comparison between different competition levels. PLoS ONE.

[40] Kulecka M, Fraczek B, Mikula M, Zeber-Lubecka N, Karczmarski J, Paziewska A (2020). The composition and richness of the gut microbiota differentiate the top Polish endurance athletes from sedentary controls. Gut Microbes.

[41] Moitinho-Silva L, Wegener M, May S, Schrinner F, Akhtar A, Boysen TJ (2021). Short-term physical exercise impacts on the human holobiont obtained by a randomised intervention study. BMC Microbiol.

[42] Petersen LM, Bautista EJ, Nguyen H, Hanson BM, Chen L, Lek SH (2017). Community characteristics of the gut microbiomes of competitive cyclists. Microbiome.

[43] O’Donovan CM, Madigan SM, Garcia-Perez I, Rankin A, O’Sullivan O, Cotter PD (2020). Distinct microbiome composition and metabolome exists across subgroups of elite Irish athletes. J Sci Med Sport.

[44] Clarke SF, Murphy EF, O’Sullivan O, Lucey AJ, Humphreys M, Hogan A (2014). Exercise and associated dietary extremes impact on gut microbial diversity. Gut.

[45] Craven J, Cox AJ, Bellinger P, Desbrow B, Irwin C, Buchan J (2021). The influence of exercise training volume alterations on the gut microbiome in highly-trained middle-distance runners. Eur J Sport Sci.

[46] Grosicki GJ, Durk RP, Bagley JR (2019). Rapid gut microbiome changes in a world-class ultramarathon runner. Physiol Rep.

[47] Hampton-Marcell JT, Eshoo TW, Cook MD, Gilbert JA, Horswill CA, Poretsky R (2020). Comparative analysis of gut microbiota following changes in training volume among swimmers. Int J Sports Med.

[48] Keohane DM, Woods T, O’Connor P, Underwood S, Cronin O, Whiston R (2019). Four men in a boat: ultra-endurance exercise alters the gut microbiome. J Sci Med Sport.

[49] Karl JP, Margolis LM, Madslien EH, Murphy NE, Castellani JW, Gundersen Y (2017). Changes in intestinal microbiota composition and metabolism coincide with increased intestinal permeability in young adults under prolonged physiological stress. Am J Physiol Gastrointest Liver Physiol.

[50] Tabone M, Bressa C, García-Merino JA, Moreno-Pérez D, Van EC, Castelli FA (2021). The effect of acute moderate-intensity exercise on the serum and fecal metabolomes and the gut microbiota of cross-country endurance athletes. Sci Rep.

[51] Zhao X, Zhang Z, Hu B, Huang W, Yuan C, Zou L (2018). Response of gut microbiota to metabolite changes induced by endurance exercise. Front Microbiol.

[52] Allen JM, Mailing LJ, Niemiro GM, Moore R, Cook MD, White BA (2018). Exercise alters gut microbiota composition and function in lean and obese humans. Med Sci Sports Exerc.

[53] Barton W, Cronin O, Garcia-Perez I, Whiston R, Holmes E, Woods T (2021). The effects of sustained fitness improvement on the gut microbiome: a longitudinal, repeated measures case-study approach. Transl Sports Med.

[54] Bycura D, Santos AC, Shiffer A, Kyman S, Winfree K, Sutliffe J (2021). Impact of different exercise modalities on the human gut microbiome. Sports Basel Switz.

[55] Cronin O, Barton W, Skuse P, Penney NC, Garcia-Perez I, Murphy EF (2018). A prospective metagenomic and metabolomic analysis of the impact of exercise and/or whey protein supplementation on the gut microbiome of sedentary adults. mSystems.

[56] Kern T, Blond MB, Hansen TH, Rosenkilde M, Quist JS, Gram AS (2020). Structured exercise alters the gut microbiota in humans with overweight and obesity-A randomized controlled trial. Int J Obes.

[57] Jung Y, Tagele SB, Son H, Ibal JC, Kerfahi D, Yun H (2020). Modulation of gut microbiota in korean navy trainees following a healthy lifestyle change. Microorganisms.

[58] Motiani KK, Collado MC, Eskelinen JJ, Virtanen KA, Löyttyniemi E, Salminen S (2020). Exercise training modulates gut microbiota profile and improves endotoxemia. Med Sci Sports Exerc.

[59] Morita E, Yokoyama H, Imai D, Takeda R, Ota A, Kawai E (2019). Aerobic exercise training with brisk walking increases intestinal bacteroides in healthy elderly women. Nutrients.

[60] Munukka E, Ahtiainen JP, Puigbó P, Jalkanen S, Pahkala K, Keskitalo A (2018). Six-week endurance exercise alters gut metagenome that is not reflected in systemic metabolism in over-weight women. Front Microbiol.

[61] Rettedal EA, Cree JME, Adams SE, MacRae C, Skidmore PML, Cameron-Smith D (2020). Short-term high-intensity interval training exercise does not affect gut bacterial community diversity or composition of lean and overweight men. Exp Physiol.

[62] Taniguchi H, Tanisawa K, Sun X, Kubo T, Hoshino Y, Hosokawa M (2018). Effects of short-term endurance exercise on gut microbiota in elderly men. Physiol Rep.

[63] Zhong F, Wen X, Yang M, Lai HY, Momma H, Cheng L (2021). Effect of an 8-week exercise training on gut microbiota in physically inactive older women. Int J Sports Med.

[64] Wei Y, Li Y, Yan L, Sun C, Miao Q, Wang Q (2020). Alterations of gut microbiome in autoimmune hepatitis. Gut.

[65] Allen JM, Berg Miller ME, Pence BD, Whitlock K, Nehra V, Gaskins HR (2015). Voluntary and forced exercise differentially alters the gut microbiome in C57BL/6J mice. J Appl Physiol.

[66] Brook I (1996). Veillonella infections in children. J Clin Microbiol.

[67] Cobo F, Pérez-Carrasco V, García-Salcedo JA, Navarro-Marí JM (2020). Bacteremia caused by Veillonella dispar in an oncological patient. Anaerobe.

[68] Rovery C, Etienne A, Foucault C, Berger P, Brouqui P (2005). Veillonella montpellierensis endocarditis. Emerg Infect Dis.

[69] Scheiman J, Luber JM, Chavkin TA, MacDonald T, Tung A, Pham LD (2019). Meta-omics analysis of elite athletes identifies a performance-enhancing microbe that functions via lactate metabolism. Nat Med.

[70] Hills RDJ, Pontefract BA, Mishcon HR, Black CA, Sutton SC, Theberge CR (2019). Gut microbiome: profound implications for diet and disease. Nutrients.

[71] Santacruz A, Collado MC, García-Valdés L, Segura MT, Martín-Lagos JA, Anjos T (2010). Gut microbiota composition is associated with body weight, weight gain and biochemical parameters in pregnant women. Br J Nutr.

[72] Ley RE, Turnbaugh PJ, Klein S, Gordon JI (2006). Microbial ecology: human gut microbes associated with obesity. Nature.

[73] Turnbaugh PJ, Bäckhed F, Fulton L, Gordon JI (2008). Diet-induced obesity is linked to marked but reversible alterations in the mouse distal gut microbiome. Cell Host Microbe.

[74] Turnbaugh PJ, Ley RE, Mahowald MA, Magrini V, Mardis ER, Gordon JI (2006). An obesity-associated gut microbiome with increased capacity for energy harvest. Nature.

[75] Tuovinen E, Keto J, Nikkilä J, Mättö J, Lähteenmäki K (2013). Cytokine response of human mononuclear cells induced by intestinal Clostridium species. Anaerobe.

[76] Rupnik M, Wilcox MH, Gerding DN (2009). Clostridium difficile infection: new developments in epidemiology and pathogenesis. Nat Rev Microbiol.

[77] Kelly CP, LaMont JT (2008). Clostridium difficile–more difficult than ever. N Engl J Med.

[78] Collins DA, Sohn KM, Wu Y, Ouchi K, Ishii Y, Elliott B (2020). Clostridioides difficile infection in the Asia-Pacific region. Emerg Microbes Infect.

[79] Burke KE, Lamont JT (2014). Clostridium difficile infection: a worldwide disease. Gut Liver.

[80] Lessa FC, Winston LG, McDonald LC (2015). Burden of Clostridium difficile infection in the United States. N Engl J Med.

[81] Bishara J, Farah R, Mograbi J, Khalaila W, Abu-Elheja O, Mahamid M (2013). Obesity as a risk factor for Clostridium difficile infection. Clin Infect Dis Off Publ Infect Dis Soc Am.

[82] Leung J, Burke B, Ford D, Garvin G, Korn C, Sulis C (2013). Possible association between obesity and Clostridium difficile infection. Emerg Infect Dis.

[83] Sokol H, Pigneur B, Watterlot L, Lakhdari O, Bermúdez-Humarán LG, Gratadoux JJ (2008). Faecalibacterium prausnitzii is an anti-inflammatory commensal bacterium identified by gut microbiota analysis of Crohn disease patients. Proc Natl Acad Sci USA.

[84] Furet JP, Kong LC, Tap J, Poitou C, Basdevant A, Bouillot JL (2010). Differential adaptation of human gut microbiota to bariatric surgery-induced weight loss: links with metabolic and low-grade inflammation markers. Diabetes.

[85] Machiels K, Joossens M, Sabino J, De Preter V, Arijs I, Eeckhaut V (2014). A decrease of the butyrate-producing species Roseburia hominis and Faecalibacterium prausnitzii defines dysbiosis in patients with ulcerative colitis. Gut.

[86] Cotillard A, Kennedy SP, Kong LC, Prifti E, Pons N, Le Chatelier E (2013). Dietary intervention impact on gut microbial gene richness. Nature.

[87] Zhernakova A, Kurilshikov A, Bonder MJ, Tigchelaar EF, Schirmer M, Vatanen T (2016). Population-based metagenomics analysis reveals markers for gut microbiome composition and diversity. Science.

[88] Hippe B, Zwielehner J, Liszt K, Lassl C, Unger F, Haslberger AG (2011). Quantification of butyryl CoA: acetate CoA-transferase genes reveals different butyrate production capacity in individuals according to diet and age. FEMS Microbiol Lett.

[89] Louis P, Duncan SH, McCrae SI, Millar J, Jackson MS, Flint HJ (2004). Restricted distribution of the butyrate kinase pathway among butyrate-producing bacteria from the human colon. J Bacteriol.

[90] Pryde SE, Duncan SH, Hold GL, Stewart CS, Flint HJ (2002). The microbiology of butyrate formation in the human colon. FEMS Microbiol Lett.

[91] Shaw KA, Bertha M, Hofmekler T, Chopra P, Vatanen T, Srivatsa A (2016). Dysbiosis, inflammation, and response to treatment: a longitudinal study of pediatric subjects with newly diagnosed inflammatory bowel disease. Genome Med.

[92] Kampmann C, Dicksved J, Engstrand L, Rautelin H (2016). Composition of human faecal microbiota in resistance to Campylobacter infection. Clin Microbiol Infect Off Publ Eur Soc Clin Microbiol Infect Dis.

[93] Turroni F, Marchesi JR, Foroni E, Gueimonde M, Shanahan F, Margolles A (2009). Microbiomic analysis of the bifidobacterial population in the human distal gut. ISME J.

[94] Rerksuppaphol S, Rerksuppaphol L (2015). A randomized double-blind controlled trial of lactobacillus acidophilus plus bifidobacterium bifidum versus placebo in patients with hypercholesterolemia. J Clin Diagn Res JCDR.

[95] Sharma P, Bhardwaj P, Singh R (2016). Administration of Lactobacillus casei and Bifidobacterium bifidum Ameliorated Hyperglycemia, dyslipidemia, and oxidative stress in diabetic rats. Int J Prev Med.

[96] Lomax AR, Calder PC (2009). Probiotics, immune function, infection and inflammation: a review of the evidence from studies conducted in humans. Curr Pharm Des.

[97] Salminen S, Nybom S, Meriluoto J, Collado MC, Vesterlund S, El-Nezami H (2010). Interaction of probiotics and pathogens–benefits to human health?. Curr Opin Biotechnol.

[98] Aizawa E, Tsuji H, Asahara T, Takahashi T, Teraishi T, Yoshida S (2016). Possible association of Bifidobacterium and Lactobacillus in the gut microbiota of patients with major depressive disorder. J Affect Disord.

[99] Ley RE (2016). Gut microbiota in 2015: prevotella in the gut: choose carefully. Nat Rev Gastroenterol Hepatol.

[100] Alauzet C, Marchandin H, Lozniewski A (2010). New insights into Prevotella diversity and medical microbiology. Future Microbiol.

[101] Mörkl S, Lackner S, Meinitzer A, Mangge H, Lehofer M, Halwachs B (2018). Gut microbiota, dietary intakes and intestinal permeability reflected by serum zonulin in women. Eur J Nutr.

[102] Chen YR, Zheng HM, Zhang GX, Chen FL, Chen LD, Yang ZC (2020). High oscillospira abundance indicates constipation and low bmi in the guangdong gut microbiome project. Sci Rep.

[103] Tims S, Derom C, Jonkers DM, Vlietinck R, Saris WH, Kleerebezem M (2013). Microbiota conservation and BMI signatures in adult monozygotic twins. ISME J.

[104] Goodrich JK, Waters JL, Poole AC, Sutter JL, Koren O, Blekhman R (2014). Human genetics shape the gut microbiome. Cell.

[105] Zhai Q, Feng S, Arjan N, Chen W (2019). A next generation probiotic, Akkermansia muciniphila. Crit Rev Food Sci Nutr.

[106] Cani PD, de Vos WM (2017). Next-generation beneficial microbes: the case of akkermansia muciniphila. Front Microbiol.

[107] Org E, Parks BW, Joo JWJ, Emert B, Schwartzman W, Kang EY (2015). Genetic and environmental control of host-gut microbiota interactions. Genome Res.

[108] Everard A, Belzer C, Geurts L, Ouwerkerk JP, Druart C, Bindels LB (2013). Cross-talk between Akkermansia muciniphila and intestinal epithelium controls diet-induced obesity. Proc Natl Acad Sci U S A.

[109] Everard A, Lazarevic V, Gaïa N, Johansson M, Ståhlman M, Backhed F (2014). Microbiome of prebiotic-treated mice reveals novel targets involved in host response during obesity. ISME J.

[110] Derrien M, Belzer C, de Vos WM (2017). Akkermansia muciniphila and its role in regulating host functions. Microb Pathog.

[111] Przewłócka K, Folwarski M, Kaźmierczak-Siedlecka K, Skonieczna-Żydecka K, Kaczor JJ (2020). Gut-muscle axisexists and may affect skeletal muscle adaptation to training. Nutrients.

[112] Ticinesi A, Lauretani F, Tana C, Nouvenne A, Ridolo E, Meschi T (2019). Exercise and immune system as modulators of intestinal microbiome: implications for the gut-muscle axis hypothesis. Exerc Immunol Rev.

